# Supplementary material for: Chromatin opening ability of pioneer factor Pax7 depends on unique isoform and C-terminal domain
Source: Nucleic Acids Res. 2023 Jun 16;51(14):7254–68. doi: 10.1093/nar/gkad520 (PMC10415112; doi:10.1093/nar/gkad520)
Supplement: gkad520_Supplemental_Files [file gkad520_supplemental_files.zip › Bascunana Supplementary Data.pdf]

Chromatin opening ability of pioneer factor Pax7 depends on unique isoform and C-terminal domain

Legends to Supplementary Figures

Supplementary Figure 1. Characterization of Pax7 isoforms expressed in AtT-20 cells.

a) Schematic representation of the Pax7 DNA-binding Paired domain with its two helix-turn-helix subdomains (PAI and RED). The position of residues Q108 and GL151/152 are shown relative to the six α helices of the Paired domain.

b) Western Blot analysis of pools of AtT-20 cells (left) expressing each Pax7 isoform (arrowhead) and showing similar levels of Pax7 protein compared to PCNA control (arrow). Comparison of Pax7 expression in mouse neurointermediate pituitary (NIL) with the AtT-20-Pax7 pool of stably transfected (retroviral vector) cells that express a larger Flagged (Pax7-FL) version of Pax7; quantitation relative to GAPDH is shown at bottom. The NIL tissue extract is about 50% Pax7-expressing melanotropes (the remainder being mostly posterior lobe glial cells). The AtT-20 Pax7 cells thus express about 1,9 times more Pax7 compared to normal mouse pituitary melanotropes.

c) PCA plot analysis of transcriptome data for RNA-Seq duplicates of AtT-20 cells expressing each Pax7 isoform and Pax3.

d) Violin plot comparisons of differentially expressed genes (RNA-Seq) for the indicated isoform compared to the reference isoform Pax7 Q+GL-.

e) Volcano Plot representation of differentially expressed genes in AtT-20 cells expressing the reference Pax7 Q+GL- isoform compared to control Neo cells.

f) Volcano Plot representation of differentially expressed genes in AtT-20 cells expressing the Q+GL+ isoform compared to control Neo cells.

g) Transcriptional activity of Pax7 isoforms assessed by co-transfection with luciferase reporters driven by trimers of the indicated Pax7 binding sites inserted upstream of the minimal POMC promoter.

Supplementary Figure 2. Characterization of ChIP-Seq replicates for Pax7 and its isoforms expressed in AtT-20 cells.

a) Comparison of Pax7 ChIP-Seq signals for replicates of each Pax7 isoforms.

b) Comparisons of ChIP-Seq signals for each isoform compared to replica 1 of Pax7 reference isoform Q+GL-.

c) Means fold changes by site categories for each Pax7 isoform.

Supplementary Figure 3. Isoform specific chromatin opening correlates with melanotrope transcriptome.

a-c) Pioneer ability of the two pituitary-expressed Pax7 isoforms assessed by ATAC-Seq (**a**), Pax7 ChIP-Seq (**b**) and H3K4me1 ChIP-Seq (**c**). Heatmaps are shown for the subsets of Pioneer, Activated and Constitutive enhancer sites. The Pioneer subset is subdivided in two subsets of GL-sensitive (ATAC) and GL- insensitive (ATAC) based on the ATAC-Seq profiles.

d) Comparisons of Pax7 ChIP-Seq strength (reads density) for the two subsets of GL-sensitive and GL-insensitive pioneered sites.

e) Distribution of ATAC-Seq signals for Pax7 isoforms at the GL-sensitive and GL-insensitive Pioneer sites.

f) Frequencies of Pax7 binding motifs present in each subset of Pax7 Pioneer sites.

g) Total number of Pax7 binding motifs per subset of Pioneer sites.

Supplementary Figure 4. Motif analysis for the GL related Pax7 Pioneer sites. Searches were conducted for de novo and known DNA binding motifs as indicated.

Supplementary Figure 5. Read density plots and correlations between marks at Pioneer sites.

a) Box plot comparisons of the Pax7 GL- and GL+ isoforms for recruitment of Pax7, Tpit, Ash2, Brg1 measured by ChIP-Seq (reads density) at the different subsets of GL-sensitive and GL-insensitive Pioneer sites.

b) Top -Correlation between the recruitment of the Pax7 isoform Q+GL+ and recruitment of the nonpioneer factor Tpit, Ash2 and Brg1 at Pioneer sites (reads density). Bottom- Correlation between the recruitment of Tpit and the remodeling factors Ash2 and Brg1 at Pioneer sites for the Pax7 isoform Q+GL+ (reads density).

Supplementary Figure 6. Weak correlations of Pax7 ChIP-Seq signals for the GL- (left) and GL+ (right) isoforms compared with H3K4me1 (middle) and ATAC-Seq signals (bottom).

Supplementary Figure 7. Structure function investigation of Pax7.

a) Sequence comparison of mouse Pax3 with Pax7. Paired and HD domains are highlighted in blue and green respectively, and the endpoints of C-terminal deletions indicated by red bar. The OAR domain is in purple. Yellow and light blue boxes indicate residues mutated to alanine.

b) Western Blot analyses of Pax7 C-terminal deletion-e mutants expressed in AtT-20 cells compared to PCNA control.

c-d) Assessment of different alanine mutations in the interval between aa431-465 of Pax7-465 for pioneer and transcriptional activity upon expression in AtT-20 cells. Genomic recruitment of the different mutants was assessed by ChIP-qPCR (**c**) and target gene expression by RT-qPCR (**d**) at a locus requiring enhancer opening by pioneer action (*Pcsk2*) and at loci where Pax7 acts only as a transcriptional activator (*Pde2a* and *Lmcd1*).

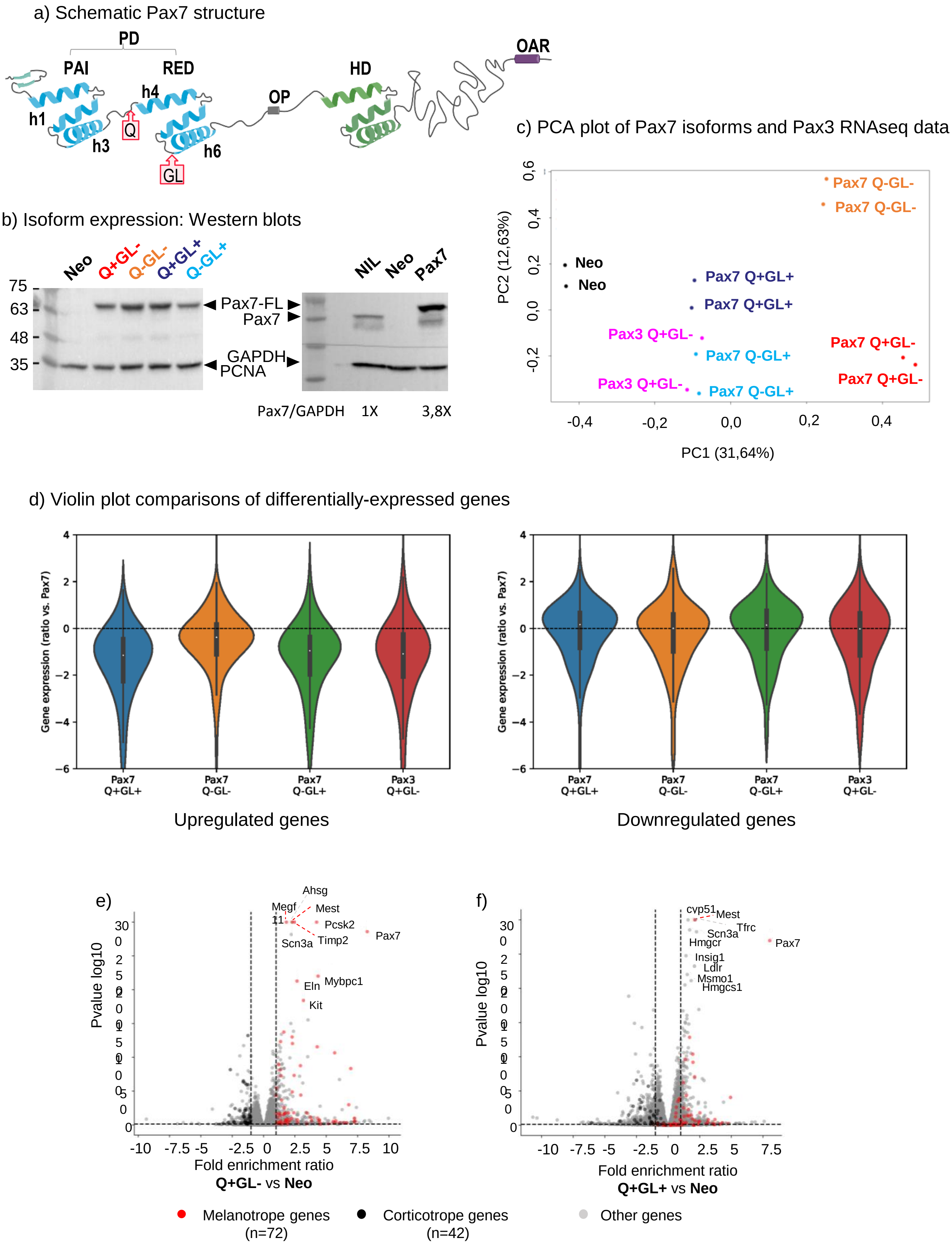

Supplementary Figure 1. RNAseq reproducibility and transcriptional activity

g) Transcriptional activity of Pax7 isoforms assessed with Luciferase reporters for the indicated DNA motifs

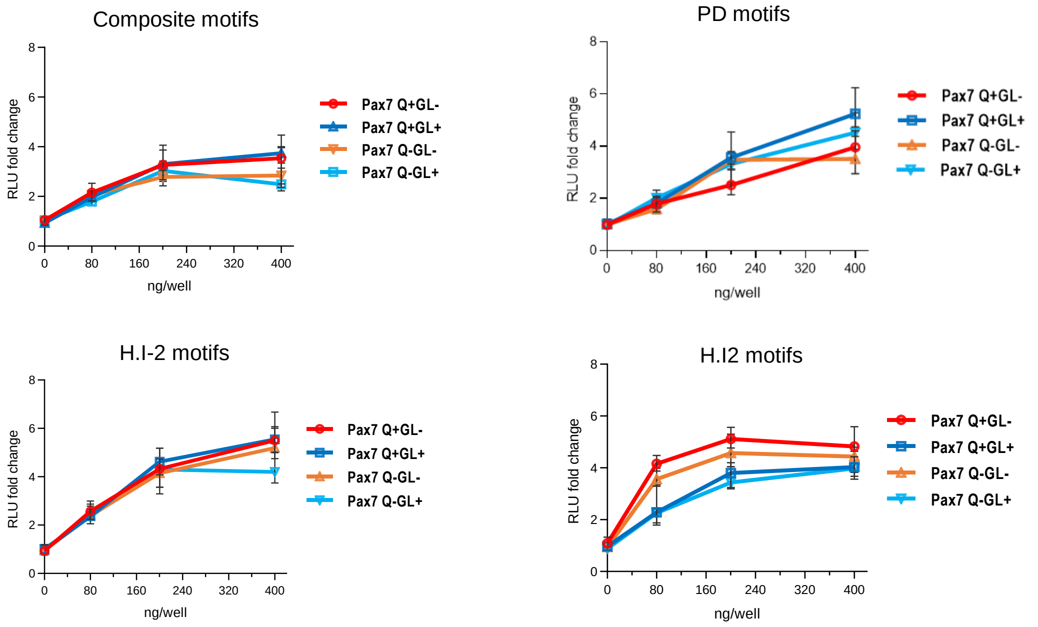

Supplementary Figure 1. RNAseq reproducibility and transcriptional activity

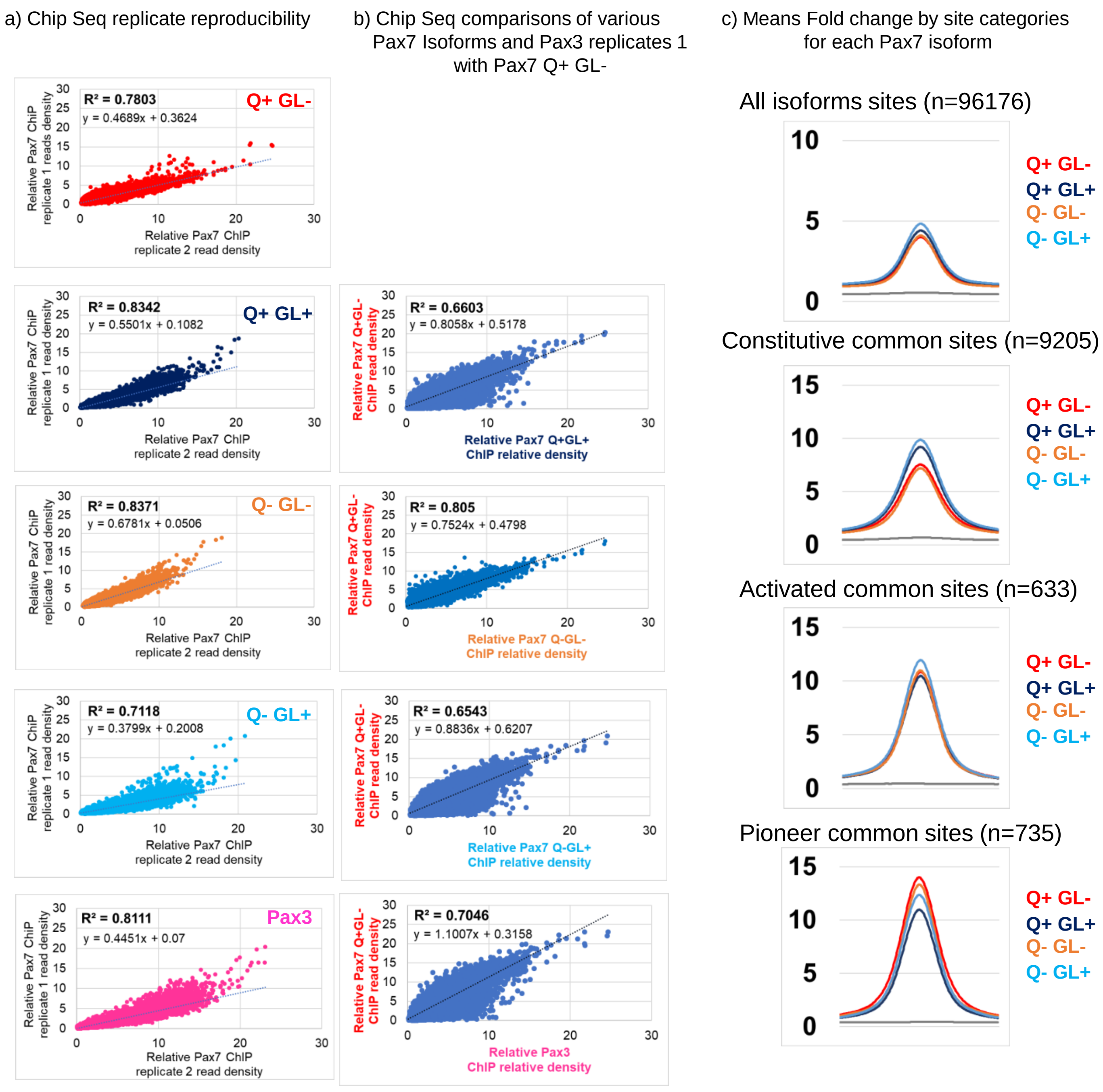

Supplementary Figure 2. ChIPseq reproducibility

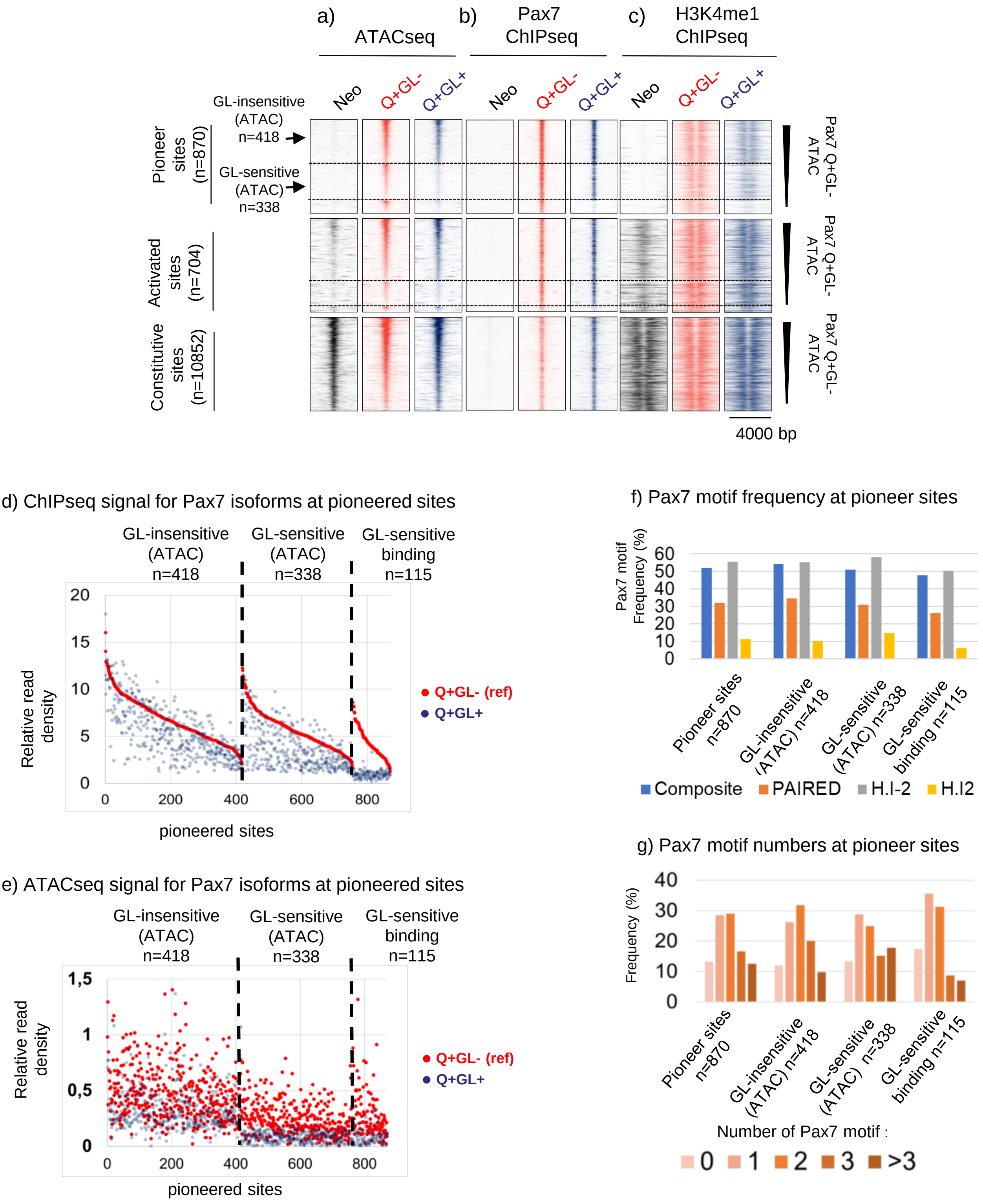

Supplementary Figure 3. Isoform-specific chromatin opening correlates with melanotrope transcriptome

GL-insensitive (H3K4me1) n=242

De novo motifs

Rank	Motif	P-value	% of Targets	% of Background	
1	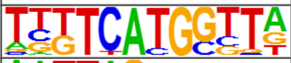	1e-44	44.21%	9.41%	PAIRED related
2	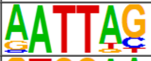	1e-28	78.51%	43.23%	H.I2
3	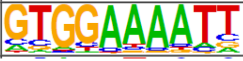	1e-25	34.71%	9.63%	NFAT

Known motifs

Rank	Motif	Name	P-value	% of Targets Sequences with Motif	% of Background Sequences with Motif	
1	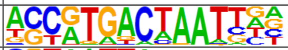	PAX3:FKHR-fusion(Paired,Homeobox)/Rh4-PAX3:FKHR-ChIP-Seq(GSE19063)/Homer	1e-27	20.66%	2.80%	Composite
2	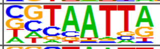	DLX5(Homeobox)/BasalGanglia-Dlx5-ChIP-seq(GSE124936)/Homer	1e-16	30.99%	11.04%	H.I2
3	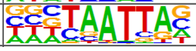	DLX2(Homeobox)/BasalGanglia-Dlx2-ChIP-seq(GSE124936)/Homer	1e-14	45.45%	22.24%	H.I2

GL-sensitive (H3K4me1) n=259

De novo motifs

Rank	Motif	P-value	% of Targets	% of Background	
1	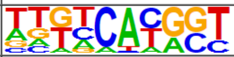	1e-56	55.60%	13.47%	PAIRED related
2	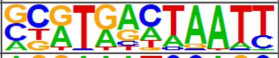	1e-39	32.05%	5.43%	Composite
3	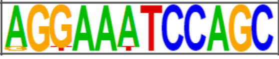	1e-29	4.25%	0.01%	RBM6

Known motifs

Rank	Motif	Name	P-value	% of Targets Sequences with Motif	% of Background Sequences with Motif	
1	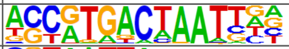	PAX3:FKHR-fusion(Paired,Homeobox)/Rh4-PAX3:FKHR-ChIP-Seq(GSE19063)/Homer	1e-37	24.32%	2.93%	Composite
2	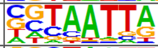	DLX5(Homeobox)/BasalGanglia-Dlx5-ChIP-seq(GSE124936)/Homer	1e-22	33.59%	10.73%	H.I2
3	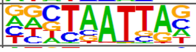	En1(Homeobox)/SUM149-EN1-ChIP-Seq(GSE120957)/Homer	1e-22	54.44%	25.65%	H.I2

GL-sensitive binding n=115

De novo motifs

Rank	Motif	P-value	% of Targets	% of Background	
1	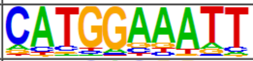	1e-18	49.57%	14.39%	NFAT
2	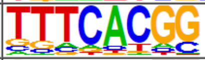	1e-15	61.74%	25.48%	PAIRED related
3	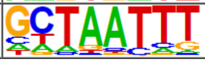	1e-12	55.65%	24.04%	H.I2

Known motifs

Rank	Motif	Name	P-value	% of Targets Sequences with Motif	% of Background Sequences with Motif	
1	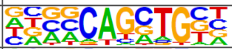	Ascl1(bHLH)/NeuralTubes-Ascl1-ChIP-Seq(GSE55840)/Homer	1e-14	46.96%	15.92%	BHLH
2	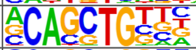	Ptf1a(bHLH)/Panc1-Ptf1a-ChIP-Seq(GSE47459)/Homer	1e-13	63.48%	29.36%	BHLH
3	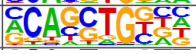	HEB(bHLH)/mES-Heb-ChIP-Seq(GSE53233)/Homer	1e-12	48.70%	18.85%	BHLH

Supplementary Figure 4. Motif analysis for the GL related sites

a) Read density plots of activating marks at pioneer enhancers

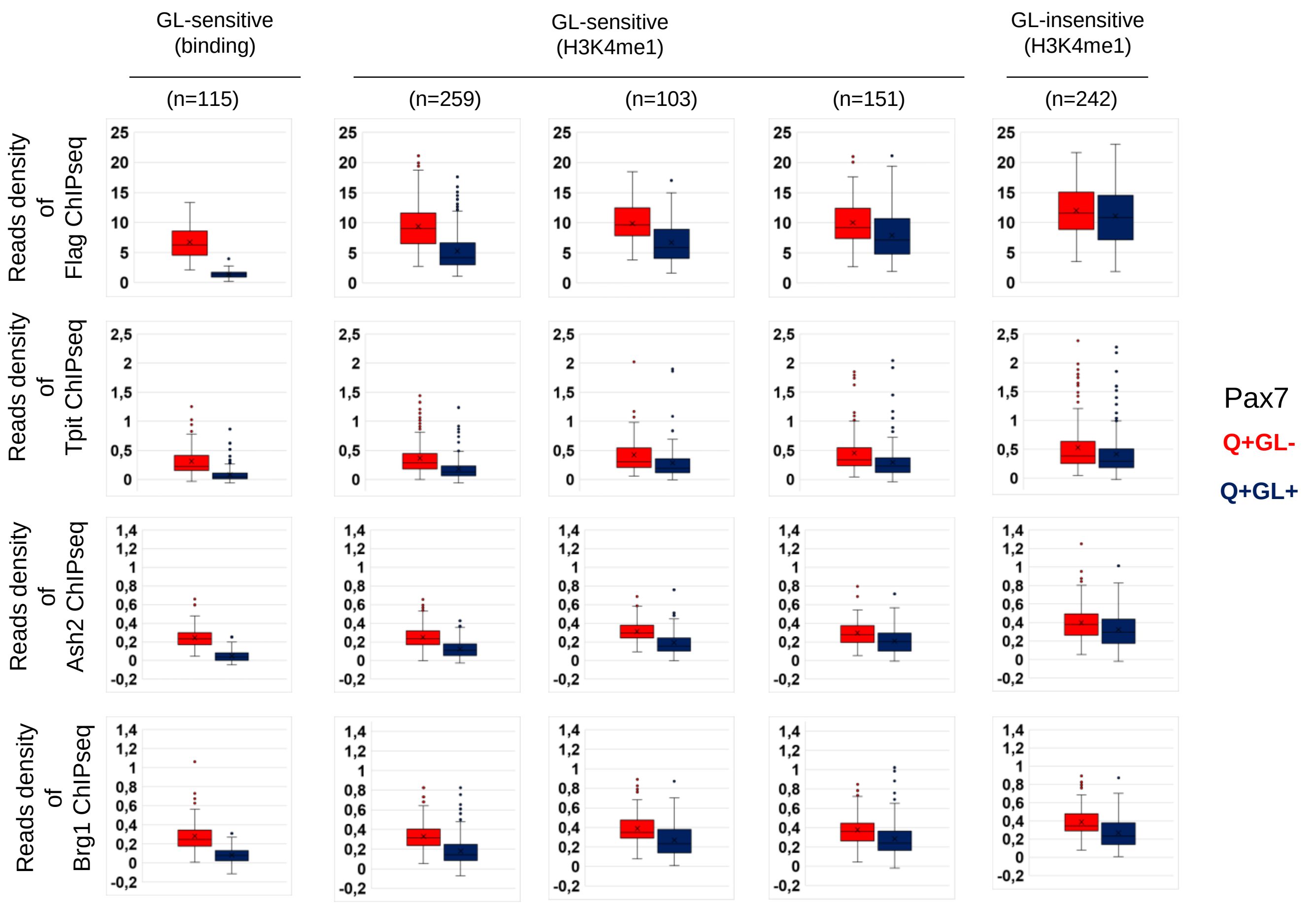

b) Correlation between activating marks and transcription factor recruitments

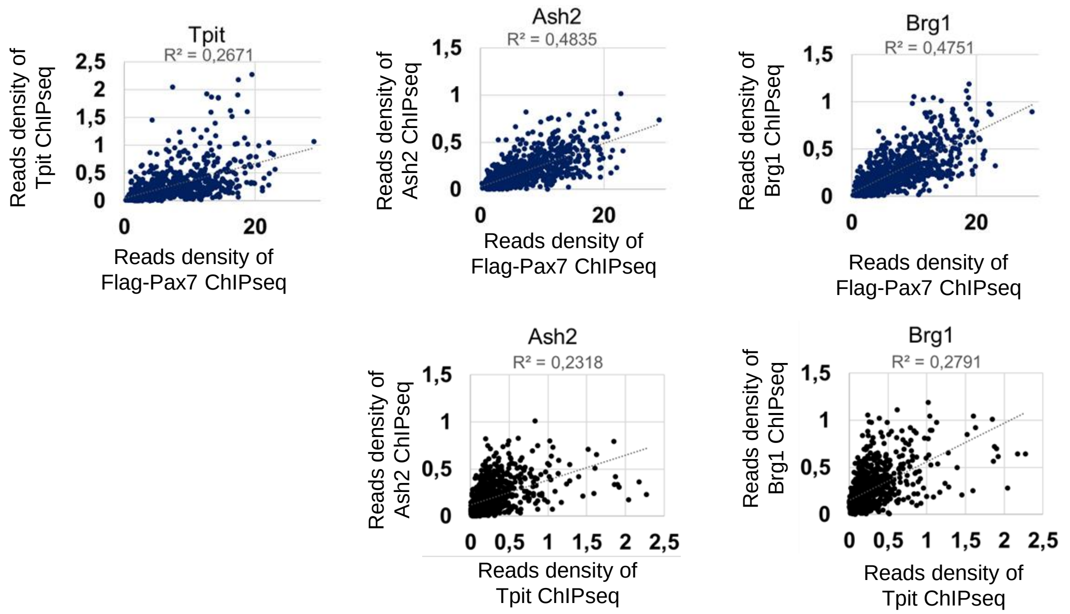

Supplementary Figure 5. Read density plots and correlations between marks at pioneer sites

a) Correlation of Pax7 recruitment for pituitary isoforms on common pioneer sites (n=755)

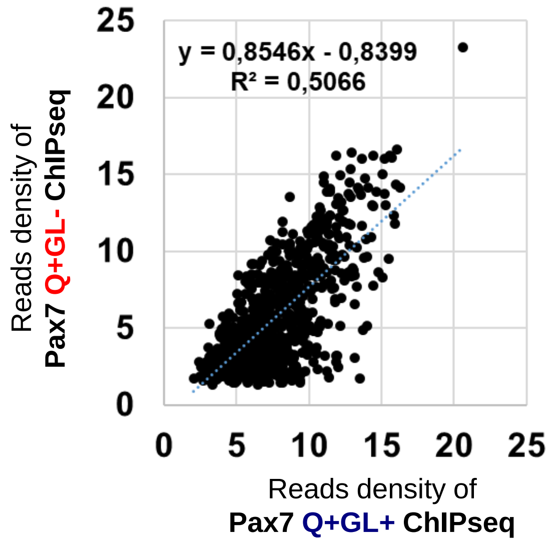

b) Correlation between H3K4me1 signals and Pax7 recruitments on common pioneer sites

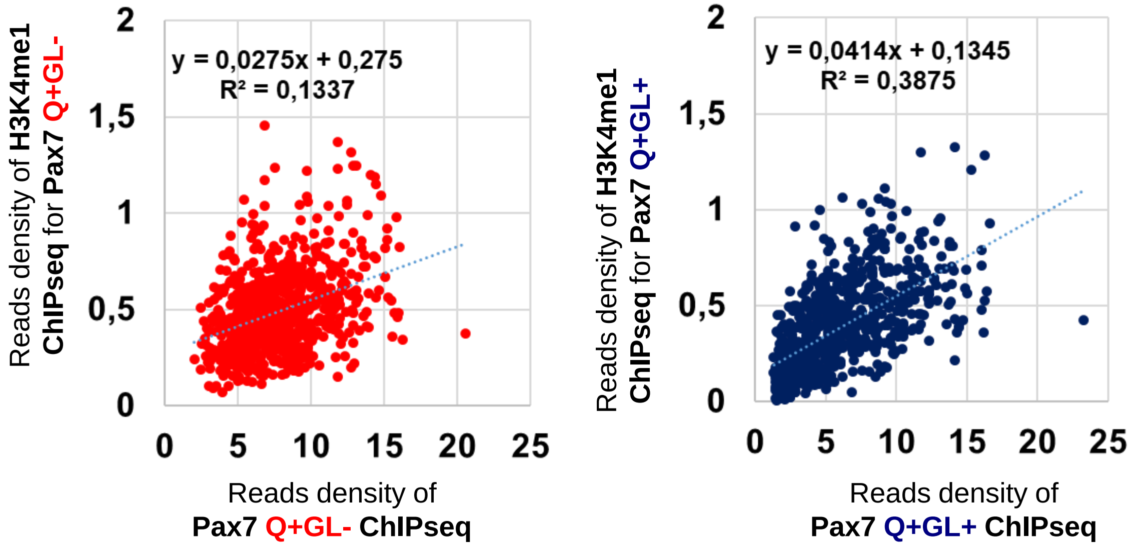

c) Correlation between ATACseq signals and Pax7 recruitments on common pioneer sites

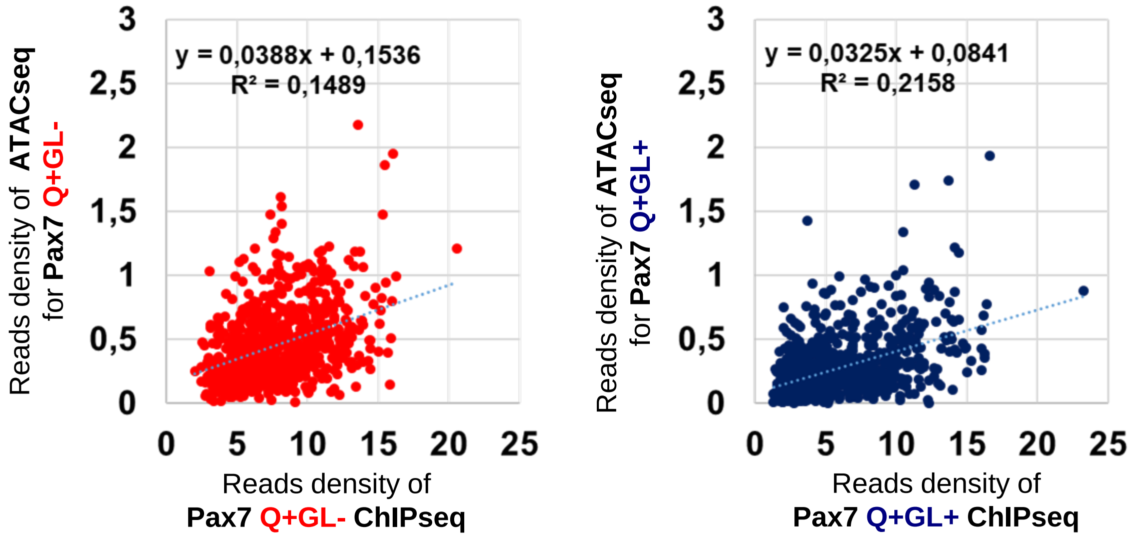

Supplementary Figure 6. Weak correlations between Pax7 GL- or Pax7 GL+ (as indicated) recruitment strength and corresponding signals for H3K4me1 ChIP-Seq and ATAC-Seq.

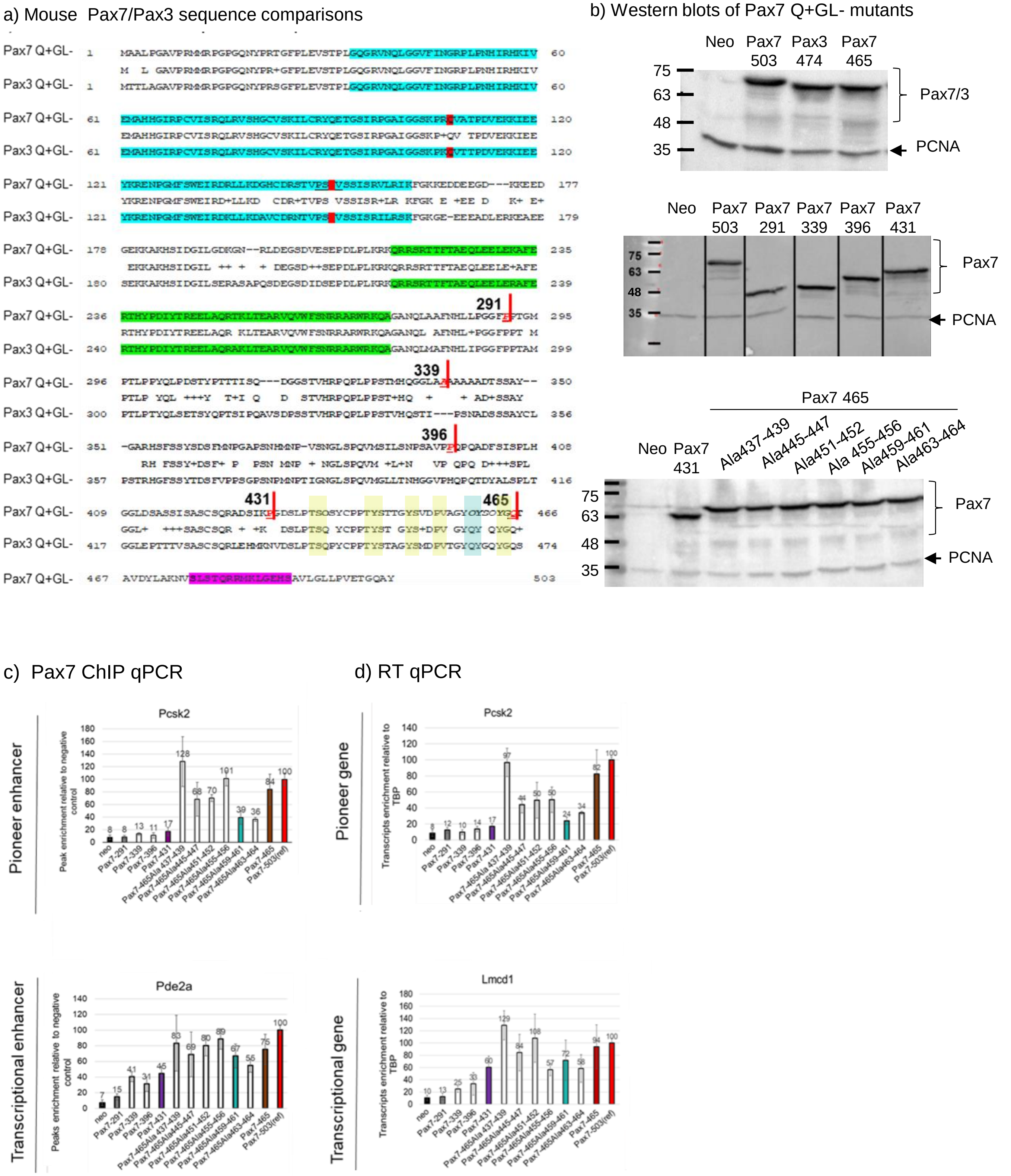

Supplementary Figure 7. Structure function study of Pax7
